# Sex difference in survival status among antiretroviral therapy users in Yirgalem general hospital, Sidama zone, south nations nationalities and peoples’ region (SNNPR), Ethiopia: retrospective cohort study

**DOI:** 10.1186/s12889-019-7672-6

**Published:** 2019-10-22

**Authors:** Mahilet Berhanu Habte, Gurmesa Tura Debelew, Tsedach Alemu Abebe

**Affiliations:** Department of Population and Family Health, Institute of Health, Jimma University, P.O.Box: 378, Jimma, Ethiopia

**Keywords:** HIV/AIDS, Survival status, Antiretroviral therapy, Gender difference, Yirgalem general hospital, Ethiopia

## Abstract

**Background:**

Antiretroviral treatment (ART) has been shown to enhance the survival of people living with HIV worldwide. In Ethiopia, the number of ART users has increased from 47,422 in 2005 to 703,516 in 2017; yet, early mortality of patients has presented challenges to the success of the ART program. Because of gender roles, it is assumed that females are at risk of dying earlier after the start of the medications. Hence, this study aimed to assess the sex difference in the survival status among the ART users.

**Methods:**

A retrospective cohort study was conducted in March 2017 among sample of 687 ART users registered from 2010 to 2015. Data were extracted from patient records by using a structured checklist. The extracted data were analyzed by STATA version 13. Survival analysis and Cox regression were used to determine survival status and identify associated factors.

**Results:**

Among 685 reviewed records of ART users, 20 males and 64 females died in the 5 years period of ART initiation. This makes the overall 5 years survival rate of 84.23%. Females had lower survival probability (80.10%) as compared to males (91.18%) (Adjusted Hazard Ratio (AHR) = 1.79; 95% CI: 1.04, 3.06). Divorced individuals as compared to married (AHR = 2.09; 95% CI: 1.10, 3.97), individuals with less education (AHR = 2.54 95% CI: 1.29, 4.98) or those who attended only primary education (AHR = 2.07; 95% CI: 1.18, 3.65) as compared to those who attended secondary or above had low survival probability. Those who never disclosed their HIV status (AHR = 3.62; 95% CI: 1.25, 10.46) as compared to disclosed, bedridden individuals as compared to normal functional status (AHR = 2.7; 95% CI: 1.24, 5.89) and those who had tuberculosis (TB)-co infection (AHR = 2.60; 1.48, 4.45) had lower rates of survival.

**Conclusion:**

Females were at higher risk of dying within 5 years of ART initiation as compared to males. Hence, intervention to further reduce mortality should take sex differences into account. Behavioral interventions and HIV counseling service should also be strengthened to improve rate of disclosure and functional status as well as reduce TB co-infections.

## Background

Antiretroviral treatment (ART) has been shown to enhance the survival of people living with HIV (PLWHIV) worldwide. The goal of ART initiation has also expanded to include not only prevention of Acquired Immuno Deficiency Syndrome (AIDS)-related morbidity and mortality but also prevention of HIV transmission. However, despite the ongoing ART scale up, HIV transmission and AIDS-related mortality remain high in many parts of the world [1].

Globally, in 2017, about 21.7 million PLWHIV were accessing ART and of all adults living with HIV, 59% were accessing ART. Similarly, over the past decade, ART programs have been scaled up dramatically in the sub Saharan region and the number of ART users has increased dramatically from about 100,000 in 2004 to 15.4 million in 2017, making the ART coverage of 60% [2] However, the existing evidences show that the survival probability in this region is shorter [3, 4]. For instance in 2013, the estimated 1 year and 5 years survival of ART users in this region was about 87% (95% CI: 72, 94%) and 70% (95% CI: 36, 86%), respectively [4].

Ethiopia launched its ART initiative in 2003 based on a subsidized fee based approach. With the assistance of global and national programs, ART became available free of charge in 2005. As a result, there has been a continuous increase in the number of PLWHIV accessing life-saving ART from about 47,422 in 2005, 535,069 in 2015 to about 703,516 in 2017. As a result the ART coverage increased from 53% in 2015 to 71% in 2017 [5]. However, lost to follow up and early mortality remain significant challenges for the success of the national ART program [6–8]. In 2014, ART services were available in 1047 health facilities in Ethiopia and ART coverage for adults of 15 years and above reached 79.6% in same period [9]. In 2013, about 70.3% of individuals who ever started ART were on treatment indicating challenges of adherence [10].

The possible difference in disease spectrum and prognosis of HIV infection in men and women is a major concern with conflicting reports about the effect of sex-related differences on mortality. The progression rates might differ between women and men because of biological and socioeconomic factors [11]. It is assumed that females appear not to access HIV services as often as males and also have worse treatment outcomes, including mortality, because they have less time to keep HIV outpatient appointments due to family commitments, or socioeconomic circumstances [12]. Existing data in Ethiopia also show that the proportion of males enrolled in ART programs is lower than females [13].

Mortality is one of the main reasons for PLWHIV’s attrition from ART programs. Mortality rates also vary based on sex difference. According to a study conducted in Tikur Anbesa Specialized Hospital, Ethiopia, female ART users had about two times higher rates of dying as compared to males [14]. In contrast, a study done in Goba hospital, Ethiopia, revealed that males had three times higher rate of dying within 5 years of ART initiation as compared to females [15].

Despite the availability of a large body of research evidence that addresses issues around HIV/AIDS treatment in Ethiopia, the level of understanding about survival status and differences based on sex as a result of HIV infection is low and inconsistent across studies. In the country, the survival probability of ART users is low and early mortality as a result of HIV/AIDS remains a major public health problem. Thus, improving understanding around survival status of males versus females is critical for progress towards reducing mortality and attaining universal health for all stated under the Sustainable Development Goal (SDG)-3, particularly target 3.7 which aims at ensuring universal access to sexual and reproductive health care service. To realize this, it is crucial to estimate mortality rates and identify predictors that affect the survival rate of ART users with a particular emphasis on sex difference.

## Methods

### Study area, period and design

This study was conducted in Yirgalem General Hospital in March 2017, using a retrospective cohort study design from ART users’ data from September 2010 to August 2015. Yirgalem Hospital is one of the oldest hospitals found in South Nations and Nationalities People’s Region (SRRPR) of Ethiopia, located in Yirgalem Town at 45 km south of Hawassa city, the capital of the region. The hospital offers ART services and voluntary counseling and testing of HIV. Since 2013, it has been one of the treatment initiating center for multidrug resistant tuberculosis (TB) in the Southern part of Ethiopia. During the time of data collection, there were about 2015 adult ART users in the hospital.

### Population, sample size and sampling methods

The study population included adult ART users enrolled (i.e. started ART regimen either at the hospital or started elsewhere and referred in) from September 1, 2010 to August 31, 2015. The required sample size for this study was determined by using STATA for windows version 13 based on the following assumptions. The outcome variable was survival status and the main objective was to examine sex difference in survival status. Based on this, the estimated adjusted hazard ratio of females was 1.82 with standard deviation of 0.23. In addition, 95% level of confidence and 90% power were considered. Finally, by adding 10% probability of withdrawals and non-retrieval rate, the required sample size included 687 ART users. By using proportional allocation to size, we included the medical records of 414 females and 273 males in the follow up period.

Records of ART users were stratified first based on enrollment year and then by sex. Finally, a computer generated simple random sampling technique was used by using SPSS to select ART users’ records to be included in the study by using their unique ART identification number. The 5 years survival rate was calculated for those who have the 5 years surviving potential. It is the population enrolled from 2010 to 2011 and other ART users were considered as censored (Table 1).

**Table 1:** Number of enrolled ART users and number of death by year among ART users of Yirgalem Hospital, Ethiopia, 2010–2015

Year of enrolment (ART start)	Enrolled	Death/event
2010–2011	163	19
2011–2012	138	15
2012–2013	174	18
2013–2014	128	12
2014–2015	82	20

### Data collection tool, procedure and quality control

We used a structured checklist that was prepared based on ART patient monitoring chart to extract ART users’ information from the selected records. Two experienced clinical nurses extracted the data and one BSc degree holder supervised the data collectors. Data quality was controlled by designing appropriate data collection materials, training of data collectors and supervisor and careful data entry.

### Data management and analysis

Data were coded and entered into EPIDATA version 3.1 to minimize logical errors and design skipping patterns and then, exported to STATA-SE for windows version 13 for cleaning, and analysis. Descriptive statistics such as proportions, means, medians, and standard deviation (±SD) were computed for categorical and continuous variables as needed.

Kaplan Meier (KM) survival function was run to estimate the probability of survival of ART users for both sex categories. The 5 years’ survival rate was calculated for those who had a 5 year potential of surviving and other ART users were considered as censored. Log-rank test was used to test the existence of the statistically significant difference in the KM curves. Then, 6, 12, 24, 36, 48, 54 and 60 months’ probability of survival were determined using a life table.

To identify predictors of survival status, we first conducted a univariate Cox regression analysis to estimate unadjusted Hazard Ratios (HRs). Each independent variable having *P* < 0.25 in univariate analysis was considered as a candidate for the multivariable Cox regression model. We then performed multivariable Cox regression analysis to test the existence of statistically significant association between the candidate variables and the survival status at *P* < 0.05. Adjusted Hazard ratio with 95% confidence interval was used to measure the existence of significant association and strength between predictor variables and the outcome. Assumption of proportional hazard model was checked using Schoenfeld residual plot and Goodness of fit test using global test at *p* > 0.05.

## Results

### Retrieval rate

We included 687 records of adult ART users (414 females and 273 males). However, after excluding incomplete records, a total of 685 ART users’ records were included in the analysis, comprising 412 female and 273 male ART users’ information (99.7% retrieval rate).

### Socio-demographic and psychosocial characteristics

As extracted from the ART monitoring chart, the mean age of the participants at the time of ART initiation was 32.90 years (±SD 9.24), 31.75 (±SD 8.55) for females and 34.65 (±SD 9.96) for males. Three hundred thirty (48.18%) ART users were married (59.09% of females and 40.91% of males). Two hundred forty two (35.33%) had attended primary level education. Nearly half, 310 (45.26%), were unemployed and about-three in four, 504 (73.58%), were from urban areas (Table 2).

**Table 2:** Socio-demographic and psychosocial characteristics of ART users at the start of ART, Sep 2010 – Aug 2015, in Yirgalem General Hospital, Southern Ethiopia, Mar, 2017 (*n* = 685)

Socio demographic characteristics	Female		Male		Total (%)
	Frequency	Percent	Frequency	Percent	
Age group					
15–24	65	69.15	29	30.85	94 (13.72)
25–34	210	64.42	116	35.58	326 (47.60)
35–44	103	55.68	82	44.32	185 (27.01)
45–54	25	42.37	34	57.63	59 (8.61)
> 55	9	42.86	12	57.14	21 (3.06)
Marital status					
Never married	62	47.33	69	52.67	131 (19.12)
Married	195	59.09	135	40.91	330 (48.18)
Divorced	75	70.09	32	29.91	107 (15.62)
Widowed	80	68.38	37	31.62	117 (17.08)
Level of education					
No education	90	68.18	42	31.82	132 (19.27)
Primary	154	63.64	88	36.36	242 (35.33)
Secondary	124	57.41	92	42.59	216 (31.53)
Tertiary	44	46.32	51	53.68	95 (13.87)
Occupation					
Government employed	56	42.11	77	57.89	133 (19.42)
Self employed	66	49.25	68	50.75	134 (19.56)
Unemployed	244	78.71	66	21.29	310 (45.26)
Other^a^	46	42.59	62	57.41	108 (15.77)
Residence					
Urban	305	60.52	199	39.48	504 ((73.58)
Rural	107	59.12	74	40.88	181 (26.42)
Disclosed to anyone					
Yes	330	59.46	225	40.54	555 (81.02)
No	82	63.08	48	36.92	130 (8.98)

^a^Student, daily laborer

### Survival status of ART users

**Fig. 1:**
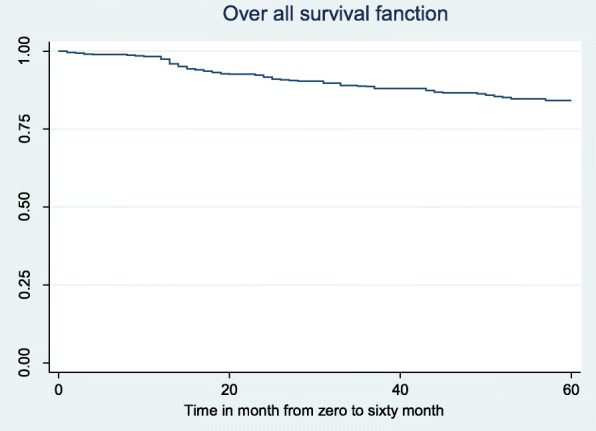
Kaplan Meier survival curve of ART users, Sep 2010 –Aug 2015, in Yirgalem General Hospital, Southern Ethiopia, Mar, 2017 (*n* = 685)

A total of 84 deaths (64 females and 20 males) were observed during the five-year treatment follow up period. The overall survival probability of ART users were 84.23% with 95% CI (80.58, 87.25%). The total follow up period included 27,209 person-months, with an overall incidence rate of 3 deaths per 1000 person-months observation. The incidence rate for females and males were 3.9 and 1.8 deaths per 1000 person-months observation, respectively (Fig. 1).

### Gender difference in survival status of ART users

**Fig. 2:**
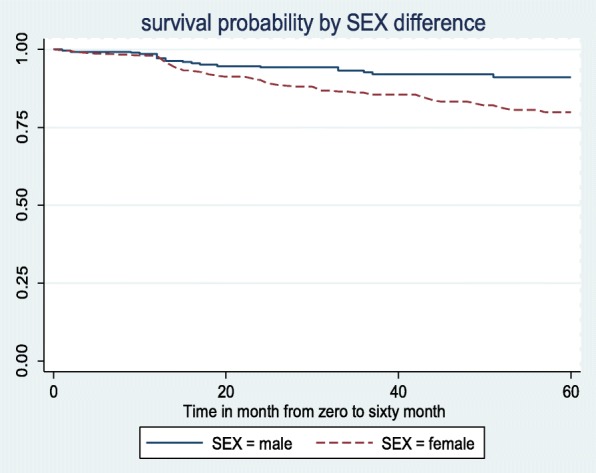
Kaplan Meier survival curve showing sex difference in survival status among ART users, Sep 2010 –Aug 2015, in Yirgalem General Hospital, Southern Ethiopia, Mar, 2017 (*n* = 685)

Probability of surviving over the 5 year treatment follow up period was 80.10% for females and 91.18% for males (AHR: 1.79, 95%CI: 1.04, 3.06) (Fig. 2).

### Determinants of survival status of ART users

To evaluate the applicability of the Cox-proportional Hazard regression model for the dataset, Schoenfeld was used. In the model, the residual plot revealed parallel plot of the observed and the residual, indicating that it is applicable for the data set. Similarly, the test of the goodness of fit of the model was not statistically significant (*P* = 0.345), indicating the model was reasonable for the data set. In addition, time dependence of the covariates was checked and all of them did not vary with time (*P* > 0.05).

Age, sex, marital status, level of education, occupation, residence, catchment area, care giver, disclosure, past history of opportunistic infection, TB co-infection at baseline, baseline AFB test result, eligibility criteria, baseline functional status, baseline World Health Organization (WHO) clinical stage, baseline weight, baseline hemoglobin level, baseline CD4 count, and cotrimoxazole prophylaxis were variables assessed in the bivariate cox regression model. However, sex, residence, educational status, marital status, catchment area, disclosure of HIV status, caregiver, baseline weight, baseline WHO stage, baseline functional status, baseline CD4 cell count, and TB co-infection at baseline had *p* < 0.25 in bivariate analysis and chosen as candidate variables for the multivariable cox regression model.

After adjustment in the multivariable cox regression analysis, sex, marital status, educational status, disclosure of HIV status, functional status and TB co-infection were found to be independent predictors of 5 years’ survival status of ART users.

Female ART users were nearly two times (AHR = 1.79; 95% CI: 1.04, 3.06) more likely to die within 5 years in the treatment follow up period compared to males. Those who were divorced had two times (AHR = 2.09; 95% CI: 1.10, 3.97) the risk of dying within 5 years of ART initiation as compared to those who were married. Those with no formal education were more than two times (AHR = 2.54; 95% CI: 1.29, 4.98) and those having only primary level education were two times (AHR = 2.07; 95% CI: 1.18, 3.65) at increased risk of dying within the 5 year treatment follow up period as compared to those with secondary and tertiary levels of education.

Those who never disclosed their HIV status to anyone had more than three times (AHR = 3.62; 95% CI: 1.25, 10.46) higher risk of dying within 5 years as compared to those who had disclosed their HIV status. Those with bedridden functional status at ART initiation had nearly three times (AHR = 2.71; 95% CI: 1.24, 5.89) higher risk of dying within 5 years as compared to those with working functional status. Those who had pulmonary TB at baseline were more than two times (AHR = 2.60; 95% CI: 1.48, 4.45) at higher risk of dying within 5 year of ART initiation as compared to those without TB co-infection (Table 3).

**Table 3:** Bivariate and Multivariable Cox-regression analysis of factors associated with survival status of ART users, Sep 2010 –Aug 2015, in Yirgalem General Hospital, Southern Ethiopia, Mar, 2017 (*n* = 685)

Variables	Level	Death (%)	Censored (%)	Unadjusted HR (95% CI)	Adjusted HR (95%CI)
Sex	Male	20 (7.3)	253(92.7)	1.00	1.00
	Female	64 (15.5)	348 (84.5)	2.14(1.29, 3.54)	1.79(1.04, 3.06)
Marital status	Married	22 (6.7)	308(93.3)	1.00	1.00
	Never married	15 (11.5)	116(88.5)	2.03(1.05, 3.91)	1.46(0.69, 3.04)
	Divorced	23 (21.5)	84 (78.5)	4.03(2.24, 7.24)	2.09(1.10, 3.97)
	Widowed	24 (20.5)	93(79.5)	3.70(2.07, 6.60)	1.46(0.72, 2.97)
Level of education	No education	32 (24.2)	100(75.8)	5.76(2.24, 14.82)	2.54(1.29, 4.98)
	Primary	28 (11.6)	214 (88.4)	2.54(0.98, 6.57)	2.07(1.18, 3.65)
	Secondary and Tertiary	24 (5.3)	287(94.7)	1.00	1.00
Disclosure	Yes	41 (7.4)	514(92.6)	1.00	1.00
	No	43 (33.1)	87 (66.9)	6.75(4.37, 10.42)	3.62(1.25, 10.46)
Functional status	Working	21 (5.3)	373 (94.7)	1.00	1.00
	Ambulatory	37 (17.6)	173(82.4)	3.33(1.95, 5.68)	1.74(0.92, 3.29)
	Bedridden	26 (32.1)	55 (67.9)	7.11(3.99, 12.64)	2.71(1.24, 5.89)
TB-co infection	No TB	32 (7.2)	414(92.8)	1.00	1.00
	INH prophylaxis	12 (10.4)	103 (89.6)	1.61(0.83, 3.13)	1.57(0.79 3.13)
	TB treatment	40 (32.3)	84(67.7)	5.50(3.45, 8.77)	2.60(1.48, 4.45)
Weight	< 40 kg	30 (26.5)	83 (73.5)	3.56(1.88, 6.72)	0.76(0.37, 1.58)
	40-60 kg	40 (10.1)	357 (89.9)	1.29(0.70, 2.37)	0.68(0.35, 1.30)
	> 60 kg	14 (8.0)	161 (92.0)	1.00	1.00
WHO stage	Stage I & II	17(4.90)	330(95.0)	1.00	1.00
	Stage III	40 (16.7)	199(83.3)	3.62(2.05, 6.39)	1.53(0.74, 3.15)
	Stage IV	27 (27.3)	72 (72.7)	7.05(3.83, 12.95)	1.77(0.74, 4.21)
CD4 count	< 200	57 (16.6)	287 (83.4)	4.10(1.48, 11.32)	2.23(0.78, 6.35)
	200–350	23 (9.9)	210 (90.1)	2.30(0.79, 6.63)	1.67(0.56, 4.96)
	> 350	4 (3.7)	104 (96.3)	1.00	1.00
Catchment	Within catchment	50 (10.8)	411 (89.2)	1.00	1.00
	Outside catchment	34 (15.2)	190 (84.8)	1.60(1.035, 2.48)	1.06(0.65, 1.70)
Caregiver	Yes	40 (7.4)	502(92.6)	1.00	1.00
	No	44 (30.8)	99(69.2)	5.99(3.88, 9.23)	1.12(.38, 3.26)
Residence	Urban	52 (10.3)	452 (89.7)	1.00	1.00
	Rural	32 (17.7)	149(82.3)	1.86(1.19, 2.88)	0.68(.38, 1.21)

## Discussion

This study highlights sex differences in survival status and its determinants among ART users in Yirgalem General Hospital, Ethiopia. Accordingly, probability of surviving for the first 5 years after ART initiation among females was 80.10%, which is significantly lower than that of males, 91.18%. This indicates that the risk of dying within 5 years of treatment follow up was nearly two times among females as compared to males. This finding is consistent with a study conducted in Tikur Anbesa Specialized hospital, Addis Ababa, Ethiopia in 2012 [14]. This might be due to the reason that females bear more work burden, including domestic work, child care and community management roles, which limit their time for rest and self-care as well as taking medications appropriately leading to early death.

However, the finding of this study were inconsistent with similar studies conducted in Goba, South East Ethiopia in 2015, in Aksum, North Ethiopia in 2014 and in Southeast Uganda in 2011 [15, 17, 18], in which males had low chance of 5 years’ survival as compared to females. This difference may be due to the reasons that males in those regions might have been engaged in risky behaviors like alcohol consumption, smoking and poor ART adherence than women.

In our study, we found that marital status had statistically significant association with the survival status of ART users. Divorced individuals were two times more at risk of dying within 5 years treatment follow up as compared to married ART users. This is in agreement with a study done in the United States in 2013 [19]. This may indicate that divorced people in the study area and in the United States may have limited social/family support and less social integration leading to poor survival as compared to those who are in a stable marital union. The existence of partner may also increase adherence and appropriate use of drugs by reminding each other.

Our study also revealed that a greater educational status increases the probability of survival for the first 5 years of ART initiation. This finding is consistent with a previous study done in Aksum Hospital Northern Ethiopia in 2014 [18]. This may be due to the reason that educated individuals have exposure to better sources of information like the internet, written materials and better understanding about the disease and the importance of ART adherence, leading to better survival.

Not disclosing HIV sero-status was also found to reduce the 5 years’ survival in this study. This finding is consistent with a study done in Jinka, South Omo, Ethiopia in 2016 [16]. Disclosing HIV status to others may reduce the risk of depression and the fear of stigma and discrimination. Individuals who disclosed their HIV status may also have better acceptance, supportive care and reminder from others about their drug schedule leading to better quality of life and survival.

In our study, we found that those who were bedridden at baseline were nearly three times more likely to die in the 5 year treatment follow up period compared to those with working functional status. This finding is consistent with similar studies conducted in Ethiopia, including the Armed Forces General Teaching Hospital (2012), Debre Marcos Referral Hospital (2014), Tikur Anbessa Specialized Hospital (2012), Hadiya and Kembata zone public health facility (2015), Jinka South Omo (2016), as well as similar analyses conducted in India [14, 16, 20–24].

Additionally, we found that individuals with TB-HIV co-infection at baseline had more than two times higher risk of dying within the 5 year treatment follow up period. This finding is consistent with studies done in other parts of the country and abroad [15–17, 24–26]. Co-infection may lead to faster deterioration of the immune system, further exacerbating patients’ clinical conditions and lead to early death [27]. The burden of drug side effects of both diseases, drug-drug interactions and adverse drug reactions might have also led to early death.

In terms of program implications, findings of this study support the current WHO and Ethiopian guidelines regarding ART initiation at early clinical stage, which is test and treat approach regardless of any criteria [28, 29]. HIV counseling and testing (HCT) is a gateway for treatment provision. Though HCT services are aimed at reaching groups at higher risk of HIV acquisition, patients are often diagnosed late in the course of HIV disease. This can result in poor quality of life and early mortality, as well as delays in achieving the Sustainable Development Goal-3, aimed at ensuring healthy live and well-being for all. This also needs improving the survival of female ART users at least as that of males by addressing the gender related social determinants.

## Limitations

This study is not without limitations. For example, mortality might have been overestimated since real causes of death were not ascertained so that all deaths were considered as AIDS-related. Further, lost-to-follow up and drop-outs were censored; however, ART users may have died after changing their residence or even at home. This might have under-estimated the true level of non-survival. As the data were extracted from ART monitoring and follow-up charts, some variables like body mass index (BMI) at the baseline, socio-economic status, levels of alcohol consumption, and substance use were not assessed which might have confounded the findings.

## Conclusion

This study found that female had a lower 5 years survival probably as compared to males following ART initiation in Yirgalem hospital, Ethiopia. Overall, drop-outs contributed to a higher attrition rate of ART users during the 5 year treatment follow up period. Marital status, educational level, disclosure of HIV status, functional status and TB co-infection at baseline were found to be independent predictors of 5 years’ survival.

Empowering women through gender interventions and mobilizing mass media on social behavioral change communication on benefit of ART adherence, disclosure of HIV status and negative impact of treatment interruptions are recommended. Strengthening counseling at initiation and ART users’ retention mechanism using reminders and follow up to trace at home when lost to follow up are also crucial. Strengthening TB prevention and control strategy, and information dissemination on treatment and its impact are also very important to reduce co-infection and improve survival.

## Data Availability

The authors agreed to provide any required data as per the guidelines of the BMC up on request.

## Abbreviations

ADR: Adverse drug reaction
AIDS: Acquired immune deficiency syndrome
ALT: Alanine aminotransferase
ART: Anti-retroviral therapy
AST: Aspartate aminotransferase
AZT: Zidovudine
BMI: Body mass index
CD4: Type of T-Lymphocyte, White Blood Cells
CI: Confidence interval
CPT: Cotrimoxazole preventive therapy
D4T: Stavudine
EDHS: Ethiopian demographic health survey
HAART: Highly Active Antiretroviral Therapy
HGB: Hemoglobin
HIV: Human immune deficiency virus
HR: Hazard ratio
INH: Isoniazid
KM: Kaplan Meier
LTF: Lost to follow up
OI: Opportunistic infections
PLWHA: People Live With HIV/AIDS
SNNPR: South Nations Nationalities People Region
TB: Tuberculosis
TDF: Tinofovir
WHO: World health organization
WT: Weight

## References

[1] Panel on Antiretroviral Guidelines for Adults and Adolescents. In: Guidelines for the Use of Antiretroviral Agents in HIV-1-Infected Adults and Adolescents. Atlanta: Department of Health and Human Services; 2016. Available at: http://aidsinfo.nih.gov/guidelines. Accessed 20 Dec 2016.

[2] UNAIDS (2018). Fact sheet global statistics 2017.

[3] UNAIDS (2015). Fact sheet global statistics on HIV/AIDS 2014.

[4] Verguet S., Lim S. S., Murray C. J. L., Gakidou E., Salomon J. A. (2012). Incorporating Loss to Follow-up in Estimates of Survival Among HIV-Infected Individuals in Sub-Saharan Africa Enrolled in Antiretroviral Therapy Programs. Journal of Infectious Diseases.

[5] Bernabas GA, Sibhatu MK, Berhanel Y (2017). Antiretroviral Therapy Program in Ethiopia Benefits From Virology Treatment Monitoring. Ethiop J Health Sci.

[6] Assefa Y, Gilks CF, Lynen L, Williams O, Hill PS, Tolera T, Malvia A, Van Damme W (2017). Performance of the Antiretroviral Treatment Program in Ethiopia, 2005–2015: strengths and weaknesses toward ending AIDS. Int J Infect Dis.

[7] Assefa Y, Kiflie A, Tesfaye D, Mariam DH, Kloos H, Edwin W (2011). Outcomes of antiretroviral treatment program in Ethiopia : retention of patients in care is a major challenge and varies across health facilities. BMC Health Serv Res.

[8] Federal HIV/AIDS Prevention and Control Office (FHAPCO) [Ethiopia] (2007). Guideline for Implementation of the Antiretroviral therapy Program in Ethiopia.

[9] Federal HIV/AIDS Prevention and Control Office (FHAPCO) [Ethiopia] (2014). HIV/AIDS Strategic Plan 2015-2020.

[10] Federal Democratic Republic of Ethiopia (FDRE) (2014). Country Progress Report on The HIV/AIDS Response.

[11] Nicastri E, Angeletti C, Palmisano L, Sarmati L, Chiesi A, Geraci A (2005). Gender differences in clinical progression of HIV-1 infected individuals during long-term highly active antiretroviral therapy 2005. AIDS.

[12] Druyts E, Dybul M, Kanters S, Nachega J, Birungi J, Ford N (2013). Male gender and the risk of mortality among individuals enrolled in antiretroviral treatment programs in Africa: a systematic review and meta-analysis. AIDS.

[13] Mocroft A, Gill M, Davidson WPN (2000). Are there gender differences in starting protease inhibitors, HAART, and disease progression despite equal access to care?. J Acquir Immune Defic Syndr.

[14] As T (2012). Survival analysis of patients under chronic HIV care and antiretroviral treatment at black lion specialized hospital. Ethiop J Heal Dev.

[15] Setegn Tesfaye, Takele Abulie, Gizaw Tesfaye, Nigatu Dabere, Haile Demewoz (2015). Predictors of Mortality among Adult Antiretroviral Therapy Users in Southeastern Ethiopia: Retrospective Cohort Study. AIDS Research and Treatment.

[16] Tachbele E, Ameni G (2016). Survival and predictors of mortality among human immunodeficiency virus patients on anti-retroviral treatment at Jinka hospital, south Omo, Ethiopia : a six years retrospective cohort study. Epidemiol Health.

[17] Amuron B, Levin J, Birunghi J, Namara G, Coutinho A, Grosskurth H (2011). Mortality in an antiretroviral therapy programme in Jinja, south-East Uganda: a prospective cohort study. AIDS Res Ther.

[18] Tadesse Kidane, Haile Fisaha, Hiruy Neway (2014). Predictors of Mortality among Patients Enrolled on Antiretroviral Therapy in Aksum Hospital, Northern Ethiopia: A Retrospective Cohort Study. PLoS ONE.

[19] Kposowa AJ (2013). Marital status and HIV/AIDS mortality : evidence from the US National Longitudinal Mortality Study. Int J Infect Dis.

[20] Abebe N, Alemu K, Asfaw T, Abajobir AA (2014). Survival status of HIV positive adults on antiretroviral treatment in Debre Markos referral hospital, Northwest Ethiopia: retrospective cohort study. Pan Afr Med J.

[21] Ayele W, Mulugeta A, Desta A, Rabito FA, Patel K, Patel A (2015). Treatment outcomes and their determinants in HIV patients on anti-retroviral treatment program in selected health facilities of Kembata and Hadiya zones, southern nations, nationalities and peoples region, Ethiopia. BMC Public Health.

[22] Bajpai RC, Chaturvedi HK, Kumar S, Pandey A (2016). Estimation of life-time survival and predictors of mortality among the people living with HIV/AIDS : a case study in Andhra Pradesh, India. Int J Community Med Public Health.

[23] Chakravarty J, Tiwary NK, Prasad SR, Shukla S, Tiwari A, Mishra RN (2014). Determinants of survival in adult HIV patients on antiretroviral therapy in eastern Uttar Pradesh: a prospective study. Indian J Med Res.

[24] Kebebew K (2012). Survival analysis of HIV-infected patients under antiretroviral treatment at the armed forces general teaching hospital, Addis Ababa, Ethiopia. Ethiop j Heal Dev.

[25] Damtew B, Mengistie B, Alemayehu T (2015). Survival and determinants of mortality in adult HIV/AIDS patients initiating antiretroviral therapy in Somali region, Eastern Ethiopia. Pan Afr Med J.

[26] Wubshet Mamo, Berhane Yemane, Worku Alemayehu, Kebede Yigzaw, Diro Ermias (2012). High Loss to Followup and Early Mortality Create Substantial Reduction in Patient Retention at Antiretroviral Treatment Program in North-West Ethiopia. ISRN AIDS.

[27] Pawlowski Andrzej, Jansson Marianne, Sköld Markus, Rottenberg Martin E., Källenius Gunilla (2012). Tuberculosis and HIV Co-Infection. PLoS Pathogens.

[28] World Health Organization (WHO) (2016). Consolidated guidelines on the use of antiretroviral drugs for treating and preventing HIV infection. Recommendations for a public health approach.

[29] Federal Ministry of Health [Ethiopia] (2017). National Guidelines for Comprehensive HIV Prevention, Care and Treatment.

